# Differences in selective pressure on *dhps *and *dhfr *drug resistant mutations in western Kenya

**DOI:** 10.1186/1475-2875-11-77

**Published:** 2012-03-22

**Authors:** Andrea M McCollum, Kristan A Schneider, Sean M Griffing, Zhiyong Zhou, Simon Kariuki, Feiko Ter-Kuile, Ya Ping Shi, Laurence Slutsker, Altaf A Lal, Venkatachalam Udhayakumar, Ananias A Escalante

**Affiliations:** 1 Program in Population Biology, Ecology, and Evolution, Emory University, Atlanta, GA, USA; 2 Malaria Branch, Division of Parasitic Diseases and Malaria, Center for Global Health, Centers for Disease Control and Prevention, Atlanta, GA, USA; 3 Atlanta Research and Education Foundation, Atlanta, GA, USA; 4 Department of Mathematics, University of Vienna, Vienna, Austria; 5 Kenya Medical Research Institute, Centre for Vector Biology and Control Research, Kisumu, Kenya; 6 Liverpool School of Tropical Medicine, Liverpool, UK; 7 School of Life Sciences, Arizona State University, Tempe, AZ, USA; 8 Center for Evolutionary Medicine & Informatics, The Biodesign Institute, Arizona State University, Tempe, AZ, USA

**Keywords:** *Plasmodium*, Malaria, Dihydrofolate Reductase, Dihydropterote synthase, Sulphadoxine-pyrimethamine, Natural selection, Selective sweep, Drug resistance

## Abstract

**Background:**

Understanding the origin and spread of mutations associated with drug resistance, especially in the context of combination therapy, will help guide strategies to halt and prevent the emergence of resistance. Unfortunately, studies have assessed these complex processes when resistance is already highly prevalent. Even further, information on the evolutionary dynamics leading to multidrug-resistant parasites is scattered and limited to areas with low or seasonal malaria transmission. This study describes the dynamics of strong selection for mutations conferring resistance against sulphadoxine-pyrimethamine (SP), a combination therapy, in western Kenya between 1992 and 1999, just before SP became first-line therapy (1999). Importantly, the study is based on longitudinal data, which allows for a comprehensive analysis that contrasts with previous cross-sectional studies carried out in other endemic regions.

**Methods:**

This study used 236 blood samples collected between 1992 and 1999 in the Asembo Bay area of Kenya. Pyrosequencing was used to determine the alleles of dihydrofolate reductase (*dhfr*) and dihydropterote synthase *(dhps) *genes. Microsatellite alleles spanning 138 kb around *dhfr *and *dhps*, as well as, neutral markers spanning approximately 100 kb on chromosomes 2 and 3 were characterized.

**Results:**

By 1992, the South-Asian *dhfr *triple mutant was already spreading, albeit in low frequency, in this holoendemic Kenyan population, prior to the use of SP as a first-line therapy. Additionally, *dhfr *triple mutant alleles that originated independently from the predominant Southeast Asian lineage were present in the sample set. Likewise, *dhps *double mutants were already present as early as 1992. There is evidence for soft selective sweeps of two *dhfr *mutant alleles and the possible emergence of a selective sweep of double mutant *dhps *alleles between 1992 and 1997. The longitudinal structure of the dataset allowed estimation of selection pressures on various *dhfr *and *dhps *mutants relative to each other based on a theoretical model tailored to *P. falciparum*. The data indicate that drug selection acted differently on the resistant alleles of *dhfr *and *dhps*, as evidenced by fitness differences. Thus a combination drug therapy such as SP, by itself, does not appear to select for "multidrug"-resistant parasites in areas with high recombination rate.

**Conclusions:**

The complexity of these observations emphasizes the importance of population-based studies to evaluate the effects of strong drug selection on *Plasmodium falciparum *populations.

## Background

The massive use of drugs for treating *Plasmodium falciparum *malaria has selected for mutations that confer resistance in endemic areas worldwide, rendering traditional anti-malarial drugs ineffective in vast regions of the globe [1,2]. Artemisinin combination therapy (ACT) is now being used in many endemic areas; however, there are concerns that mutations conferring resistance against ACT could also emerge [3,4]. Understanding such complex evolutionary processes, especially in the context of combination therapies, is a matter of great interest. Valuable information about such dynamics can be obtained by retrospectively investigating the rise of resistance against sulphadoxine-pyrimethamine (SP), a combination drug therapy that has been widely used and for which the molecular basis of resistance is well known.

SP acts as an inhibitor of the *P. falciparum *folic acid pathway, and point mutations in two genes, dihydrofolate reductase (DHFR) and dihydropteroate synthetase (DHPS), confer resistance to SP [5]. Point mutations at *dhfr *codons 50, 51, 59, 108 and 164 act synergistically to increase resistance to pyrimethamine. Of note, S108N has a low level of resistance, the double mutants N51I/S108N and C59R/S108N have moderate levels of resistance, the triple mutant N51I/C59R/S108N has a higher level, and the quadruple mutant parasite (N51I/C59R/S108N/I164L) is considered to be resistant to the effects of pyrimethamine [6,7]. Similarly, mutations at *dhps *codons 436, 437, 540, 581 and 613 act synergistically to increase the level of resistance to sulphadoxine. Simply, the mutations S436A and A437G alone confer a low level of resistance, and when in combination with K540E and/or A581G and/or A613S/T the parasite has an increased level of resistance to sulphadoxine [1,8].

The evolution of drug resistance is further complicated by the fact that resistant alleles may have multiple origins intertwined with migration patterns among *P. falciparum *populations; such complex dynamics are still poorly understood. There is compelling evidence indicating a common origin for highly resistant pyrimethamine alleles across Southeast Asia and at a few sites in Africa [9-14]; however, additional, novel low frequency lineages for the triple mutant (51I/59R/108 N) *dhfr *allele have been documented in Cameroon and also in western Kenyan [14,15]. Similarly, recent studies from sites across Africa and Asia show multiple independent origins of mutations at *dhps *[16-18]. However, the patterns for *dhps *highlight different evolutionary processes than those for *dhfr*. Thus, SP-induced selection on resistance-associated mutations may differ for the two genes and across different endemic regions. Hence, reliable estimates of selective parameters for various *dhfr *and *dhps *mutations are highly desirable.

A few studies have addressed the genetic consequences of SP drug selection, yet the temporal dynamics of mutations are rarely investigated in both loci. Indeed, patterns consistent with selective sweeps of highly resistant *dhfr *alleles have been reported in multiple populations [9,19,20], but there are only a few studies on *dhps *[14,17,18,20]. Despite the limited evidence, *dhps *shows a clear pattern of reduced diversity in multiple populations, indicating an increase in mutant alleles conferring resistance to sulphadoxine. Notably, the patterns of the selective sweeps in *dhps *and *dhfr *appear to be different, providing evidence that the strength of selection is not the same on both loci [14,20]. However, all these studies are based on cross-sectional data and measures of the strength of drug selection are limited. Attempts to infer the strength of selection have been made for *dhfr *[9,21] but such estimates focused only on the proportion of clinical failures, an indirect line of evidence that does not consider the actual frequency of resistant mutations and may lead to inaccurate predictions. A direct comparison of the selective strengths on *dhfr *and *dhps *during the early stages of the onset of clinical resistance is still missing. Indeed, estimates of drug selection have not been obtained from molecular data. Moreover, pattern of selective sweeps studied so far just indicate drug selection but the importance of linking estimates of selection parameters with the pattern of the sweep have been neglected.

Here, a population-based characterization and analysis of genetic signatures around *dhfr *and *dhps *from samples collected in western Kenya from 1992-1999 was conducted. At the time these samples were collected, SP had been exerting selective pressure on *P. falciparum *populations since the 1980s. SP was introduced in Kenya as a second-line treatment for uncomplicated malaria in 1983 and as a first-line treatment in 1999 [22,23]. However, clinical SP resistance was noted as early as 1982 [23]. Thus, this study captures some of the early events in the dynamics of drug-resistant mutations in the local *P. falciparum *population. Even before SP was chosen as a first-line treatment, all alleles in the population had *dhfr *mutations associated with pyrimethamine resistance. In contrast, sulphadoxine-sensitive alleles at *dhps *were still present while resistant double-mutant alleles were increasing in frequency. The longitudinal data, allowed inferences of the selective strengths on various mutations at *dhfr *and *dhps *based on a theoretical model tailored to *P. falciparum*. Overall, these investigations highlight the differences in selective pressures on these two loci, when the drugs were part of a combination drug therapy.

## Methods

### Study subjects

Two hundred thirty-six blood samples collected from the Asembo Bay Cohort Project, from the years 1992-1999 [24], were analysed. This study was approved by the ethical committee of (Institutional Review Board) CDC and the Kenya National Ethics Review Committee. The participants provided written informed consent. In short, this was a longitudinal study conducted between 1992 and 1999 in western Kenya, a holo-endemic area of intense transmission estimated at approximately 300 infective bites per person per year [25]. Blood samples were taken from mother-infant pairs and other siblings less than five years old once per month until the children turned five years old. Malaria parasitaemia was treated with SP.

### DNA isolation and genotyping methods

DNA was isolated from whole blood using the QIAamp^® ^DNA Mini Kit (Qiagen, Valencia, CA, USA). All samples were genotyped for *P. falciparum *mutations at *dhfr *codons 50, 51, 59, 108, and 164 and *dhps *codons 436, 437, 540, 581, and 613 by pyrosequencing as previously described [20,26].

### Microsatellite characterization

Microsatellite characterization was conducted on all samples. Samples were assayed for 18 microsatellite loci that span 138 kb on chromosome 4 around *dhfr *[9-11], 18 loci that span 138 kb on chromosome 8 around *dhps *[19], five loci on chromosome 2 that span 101 kb, and four loci on chromosome 3 that span 94 kb [20]. The microsatellites used around *dhfr *are at -89, -58, -30, -17, -10, -7.5, -5.3, -4.5, -4.4, -3.8, -1.2, -0.3, 0.2, 0.52, 1.48, 4.05, 5.87, 30.3, and 50 kb; where negative numbers refer to positions 5' to the gene and positive numbers refer to positions 3' to the gene. The microsatellites used around *dhps *are at -72.7, -34.5, -18.7, -11, -7.4, -2.8, -1.5, -0.132, 0.034, 0.5, 1.4, 6.4, 9, 16.3, 22.8, 36, 49.5, and 66.1 kb. The loci around *dhps *have been previously published [19,20]; however, it was recently brought to the authors' attention that the orientation of the microsatellite loci along chromosome 8 around *dhps *was incorrect in [20]: loci that have been reported previously as 5' to *dhps *are actually 3' and vice versa. To avoid any confusion, the corrections along with previously published positions and primers are in Additional file 1: Table S1. The correct positions of *dhps *loci have been used throughout this manuscript.

The microsatellites used on chromosome 2 are at 302, 313, 319, 380, and 403 kb. The microsatellites used on chromosome 3 are at 335, 363, 383, and 429 kb. The PCR primers for 403 kb chromosome 2 are 5'-AAATATAAATCTTCTTCTTCTTTTTT-3' (forward) and 5'-TAGAGAAATAAATATATCCAT-3' (reverse); and for 363 kb chromosome 3 are 5'-CAAAAATGAAAAATGAAAAGG-3' (forward) and 5'-TAAAGGGTGCGCATATCAAT-3' (reverse). All remaining microsatellite PCR primers are detailed in [20]. Single reaction PCR and thermal cycling conditions are detailed in [9]; and nested PCR reactions and thermal cycling conditions are detailed in [10]. PCR products were separated on Applied Biosystems 3100 capillary sequencer and scored using GeneMapper^® ^software v3.7 (Applied Biosystems, Foster City, CA, USA).

### Genetic variation per locus and allele

The genetic variation for each microsatellite locus was measured by calculating the expected heterozygosity (*H_e_*) and number of alleles per locus (*L*). *H_e _*was calculated for each locus as $H_{e}=n/\left(n-\text{1}\right)\left[\text{1}-\sum p_{i}^{2}\right]$, where *n *is the number of isolates sampled and *p_i _*is the frequency of the *i*th allele (*i = 1,...,L*). The sampling variance for *H_e _*was calculated as $\text{2}\left(n-\text{1}\right)/n^{\text{3}}\left[2\left(n-2\right)\left[\sum p_{i}^{3}-{\left(\sum p_{i}^{2}\right)}^{\text{2}}\right]\right]$[19,21]. *H_e _*was calculated using all alleles that occurred in the respective group including those in isolates that carried more than one microsatellite allele.

*H_e_*, was also calculated for microsatellite loci associated with specific *dhfr *and *dhps *mutant alleles. For *dhfr *alleles, only samples with single 'clone' infections of the respective mutant allele were used. This guarantees that the microsatellite variation is linked to the respective allele. The pattern of variation present, before the occurrence of a beneficial mutation, should be reflected by *H_e _*among wildtype alleles; however, since sensitive wildtype alleles were only present at marginal frequencies an estimate of *H_e _*could not be calculated. As a proxy to estimate the initial variation, *H_e _*was calculated among non-triple mutant *dhfr *alleles. For this estimation, all mixed infections that did not contain the 51I/59R/108 N triple mutant (e.g. an isolate with mixed codon 51I/S108N was included, but an isolate with mixed codon 51I/59R/S108N was excluded) were included. At microsatellite loci around *dhps*, *H_e _*was calculated separately among isolates that contained single infections with the 437 G/540E mutant allele, and isolates that contained single infections with the sensitive (wildtype) alleles.

### Haplotype characterization

Approximately 70% of the samples used in this study were 'multiple infections', i.e. multiple parasite lineages or genomes were present in an infection. Based on *dhfr *and *dhps *genotyping alone, 63.0% and 72.2% were multiple infections, respectively. The neutral microsatellite markers on chromosomes 2 and 3 collectively showed that 70.0% of the samples contained multiple infections, and the microsatellites around *dhfr *and *dhps *showed 73.9% and 64.5% multiple infections, respectively. A goal with this study is to present a population-based perspective and analysis of the data; thus, data from multiple infections for appropriate analyses was retained. Multiple infections are inappropriate for all of the analyses; and it is stated when data from multiple infections were excluded.

Microsatellite haplotypes are defined as a collection of sites close to the genes *dhfr *and *dhps *that had low variation and were more likely to be in linkage disequilibrium. Thus, 11 microsatellite loci spanning 11.5 kb around *dhfr *and nine loci spanning 20 kb around *dhps *were used to characterize haplotypes relative to *dhfr *and *dhps *alleles. Haplotypes were classified as different if they contained > 1 different allele across loci. Only samples without mixed infections detected by pyrosequencing were used for haplotype characterization.

### Haplotype analysis

eBURST groups haplotypes, based on a simple evolution model, which assumes that one lineage or founding haplotype reaches high frequency in the population and then starts to differentiate, producing closely related haplotypes; this is depicted as a cluster [27]. Data from the 11 microsatellite loci spanning 11.5 kb around *dhfr *and nine loci spanning 20 kb around *dhps *(as for haplotype characterization) were used to depict genetic relationships in eBURST. Only samples in which multiple infections were not detected by pyrosequencing of *dhfr *or *dhps *were used for the eBURST analysis. Since eBURST does not allow for missing data, samples with incomplete haplotypes were removed; therefore, there were fewer samples utilized for the eBURST analysis than for haplotype characterization. If multiple alleles were detected at a single microsatellite locus in a sample, the most frequent allele was used, i.e. the one that was present at the highest peak in the electropherogram.

Genetic differentiation between alleles was measured using Wright's F-statistics [28]. The statistic *F_ST _*measures genetic differentiation between populations but, here, *F_ST _was *used as a statistic to compare groups of alleles. For *dhfr *the microsatellite loci from -10 kb to 1.47 kb and for *dhps *the loci from -2.5 kb to 17.5 kb were used for the *F_ST _*analysis. *F_ST _*calculations were computed using Arlequin ver 3.01 [29]. The Excel Microsatellite Toolkit was used to format data for Arlequin [30].

Linkage disequilibrium (LD) between loci along the chromosomes and also between *dhfr *and *dhps *point mutations was assessed by using an exact test of LD [31]. Samples with multiple alleles at any locus were removed from the analysis; this was done for *dhfr*, *dhps*, and the neutral markers independently. Similarly, samples where multiple infections were detected at any site were removed from the LD analysis, testing pairs of point mutations in *dhfr *and *dhps*; this was done independently for *dhfr *and *dhps *for a given sample. Only loci or sites that showed polymorphism among the used samples were used for the analysis. Associations were tested between pairs of loci or sites by using 10,000 Monte Carlo steps in Arlequin version 3.01 [29]. To correct for multiple testing the Bonferroni-Holm correction was used.

### Measuring the strength of selection

The strength of selection on *dhfr *and *dhps *was estimated from the changes in frequency over time of the various mutant alleles at each gene. The strength of selection, *s*, of allele A compared with allele B, was defined as *1 + s *being the average relative reproductive advantage of A over B [32]. Hence, if *p_t _*and *p_t+T _*are the relative frequencies of A at times *t *and *t + T*, log *1 + s = 1/T (*log *p_t+T_/(1-p_t+T_)- *log *p_t_/(1-p_t_)) *[32].

Measurements for the frequency of the advantageous allele A were made at time *t*, *p_t_*, at different equally spaced time points (*t_k _*= k*180 days (*k = 0, 1, 2..*.)) within the six years covered by the samples. The frequency at *t_k _*was calculated from all samples that were taken between time *t_k _*and *t_k _+ 360 *days. Hence, the intervals *[t_k_, t_k _+ 360] *overlapped (sliding window). The strength of selection was obtained by performing a linear regression of the explanatory variable log *p_t_/(1-p_t_)*, where only those time points *t_k _*as regressors for which at least three triple and three non-triple mutations occurred were included. The actual strength of selection per generation is derived from the slope of the linear regression divided by the number of malaria generations per year, *N_gen_*, which was assumed to be *N_gen _*= 17.3 (i.e., one transmission cycle every three weeks, corresponding to infections throughout the whole year). More precisely, if *s *is the strength of selection, and *α *and *β *are the constant and linear regression coefficients respectively, log *p_t_/(1-p_t_) = tN_gen _*log*(1 + s)- *log *p_0_/(1-p_0_) = α+β t*. Hence, *s *= exp*(β/N_gen_)-1*.

Two double mutant *dhfr *alleles were present in the Kenyan population, and both confer a level of pyrimethamine resistance. It is not clear a priori whether selection for both double mutant alleles is equally strong; therefore, the strength of selection for 51I/108 N allele with that for 59R/108 N was compared. The strength of selection of the triple mutant allele (51I/59R/108 N) was measured over the 51I/108 N and the 59R/108 N double mutants, separately. For these measurements, only samples with single infections at these alleles (as detected by pyrosequencing) were included.

The purpose of estimating these three strengths of selection at *dhfr *is as follows. If *s_1_*, *s_2_*, and *s_3 _*denote the strength of selection of 51I/59R/108 N over 51I/108 N, 51I/108 N over 59R/108 N, and 51I/59R/108 N over 59R/108 N, then the standard haploid selection model yields 1 + s_3 _= (1 + s_2_)*(1 + s_1_).

*Dhps *single mutants were at relatively low frequency and only the 437 G/540E double mutant was found in single infections. Thus, for *dhps*, the strength of selection of double mutant alleles (jointly) was measured over all other alleles. To derive the frequencies included were all samples with 437 G/540E single infections, and all samples that did not contain the mixed codon A437G/K540E. More precisely, excluded were only those samples for which it was unclear whether they contained the 437 G/540E mutant.

The reduction of *H_e _*flanking *dhfr *and *dhps *was utilized to evaluate whether the estimates for the strengths of selection were meaningful. For this purpose, *H_e _*was compared with the analytical prediction *H_e_^pred^*, given by *H_e_^pred ^*= *H_0 _(1-p_0_^2r(1-F)/s^)*. Here, *H_0 _*is the initial expected heterozygosity, *r *denotes the recombination rate, *F *the inbreeding adjustment (*F *= 1 corresponds to complete inbreeding, and *F *= 0 to random mating), and *p_0 _*is the initial frequency of the 51I/59R/108 N or 51I/108 N allele, or of the 437 G/540E allele. As in [9-14]r = 5.88*10^-4 ^Morgans/kb and *p_0 _= 10^-4 ^*were used. Also, *F = 0.4*, which corresponds to 60-70% mixed clone infections was used. For *H_e_^pred ^*among *dhps *437 G/540E alleles, *H_e _*among wildtype alleles was used as an estimate for *H_0_*, since it should not be affected by the sweep. For *H_e_^pred ^*among 51I/59R/108 N alleles, *H_e _*among double, single mutant and wildtype alleles was used as an estimate for *H_0_*. For *H_e_^pred ^*among 51I/108 N double mutants, *H_e _*among 59R/108 N double, single mutant and wildtype alleles was used as an estimate for *H_0_*.

## Results

### Genotyping results

**Figure 1:**
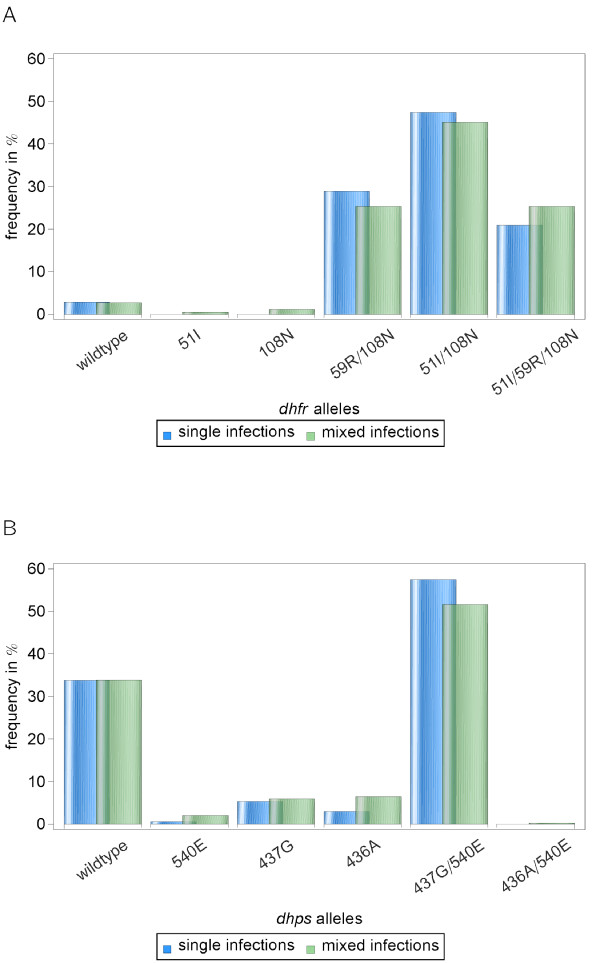
**Frequency of *dhfr *(A) and *dhps *(B) alleles in the sample set**. Bars represent the frequency in "single infections" as detected by genotyping (*dhfr *n = 145 and *dhps *n = 166), and the frequency of the alleles in "mixed infections" - single infections plus multiple infection where only one codon was a mixture of wildtype and mutant codons (*dhfr *n = 205 and *dhps *n = 217).

The frequency of *dhfr *and *dhps *alleles in the sample set was calculated with two analyses: a) including only single infections as determined by pyrosequencing of the mutations in *dhfr *or *dhps*, and b) including both the single and multiple infections that exhibited only one codon with two amino acids. For the latter, the "multiple" allele codon was used to break down the allele into two separate alleles (e.g. N51/59R/S108N was analysed as N51/59R/S108 and N51/59R/108 N). The frequency of mutant alleles is strikingly similar for the two analyses (Figure 1), but for consistency the frequency of the "single infection" sample set will be emphasized here. There were very few sensitive wildtype *dhfr *alleles in the population (3%), and the majority of the sample set is composed of double (50% 51I/108 N, 27% 59R/108 N) or triple mutant (20%) *dhfr *alleles. The majority of *dhps *alleles were sensitive wildtype (34%) and 437 G/540E mutants (57%). For both *dhfr *and *dhps*, the majority of the mutant alleles were double or triple mutant alleles.

### Haplotype analysis

In an attempt to better understand the evolutionary history of the alleles in the population, microsatellite haplotypes were characterized for the microsatellite loci immediately surrounding *dhfr *and *dhps *(Additional file 2: Figure S1 and Additional file 3: Figure S2, respectively). There were 25 haplotypes for the *dhfr *double mutant 59R/108 N (*n = *40) and 23 haplotypes for the double mutant 51I/108 N (*n = *72). The 59R/108 N allele is not dominated by any particular haplotype; however, the 51I/108 N allele is dominated by one haplotype at high frequency (haplotype 26, 64%).

Six haplotypes for the *dhfr *triple mutant (51I/59R/108 N) allele (*n = *29) were observed. Two haplotypes (15 and 17) were seen in three of the triple mutant allele samples that are also represented in allele 59R/108 N. The remaining four haplotypes (48 - 51) are not present for either of the double mutant alleles; they are unique to the triple mutant allele. Haplotype 48 is the most predominant haplotype for the triple mutant allele (79%) and is identical or closely related to the previously characterized Southeast Asian triple mutant *dhfr *haplotype [9,11]. The triple mutant *dhfr *alleles in this population also have three additional unique haplotypes, 49, 50, and 51; each present in one sample.

Haplotype analysis for *dhps *revealed 54 haplotypes for the wildtype allele (*n = *57) and 13 haplotypes for the double mutant allele 437 G/540E (*n = *95). There were no predominant haplotypes for wildtype alleles. For the samples containing the 437 G/540E allele, however, haplotype 55 was present at a high frequency (86%).

### Genetic differentiation and relationships among haplotypes and alleles

The *F_ST _*values for the comparisons between the three multiple mutant alleles of *dhfr *(51I/108 N, 59R/108 N, 51I/59R/108 N) were high and significant (p < 0.01). There was also a significant value for the comparison between the *dhps *wildtype and 437 G/540E double mutant alleles (p < 0.01).

The application eBURST was used to discern relationships among the *dhfr *and *dhps *microsatellite haplotypes. The analysis for *dhfr *was conducted by combining the data from this study with *dhfr *haplotypes previously reported from the same region of western Kenya in 38 samples collected in 2002-2004, almost 10 years after the samples used by this study were collected [15]. The majority of the *dhfr *double mutant haplotypes from 2002-2004 were present in the earlier set of samples (Additional file 4: Figure S3). A large 51I/59R/108 N cluster is comprised entirely of haplotype 48, which was the most prevalent haplotype found for the triple mutant *dhfr *allele, with the addition of rare independent haplotypes from 2002-2004 [11]. The minor frequency triple mutant allele haplotypes 17, 49, 50, and 51 are not closely related to the major frequency haplotype (haplotype 48).

An analysis of *dhps *haplotypes exhibits a single cluster comprised entirely of haplotypes from the double mutant alleles (Additional file 5: Figure S4). Specifically, all points are derived from haplotype 55, which is the haplotype in highest frequency from the 437 G/540E sample set (Additional file 3: Figure S2).

### Estimates of selection

**Figure 2:**
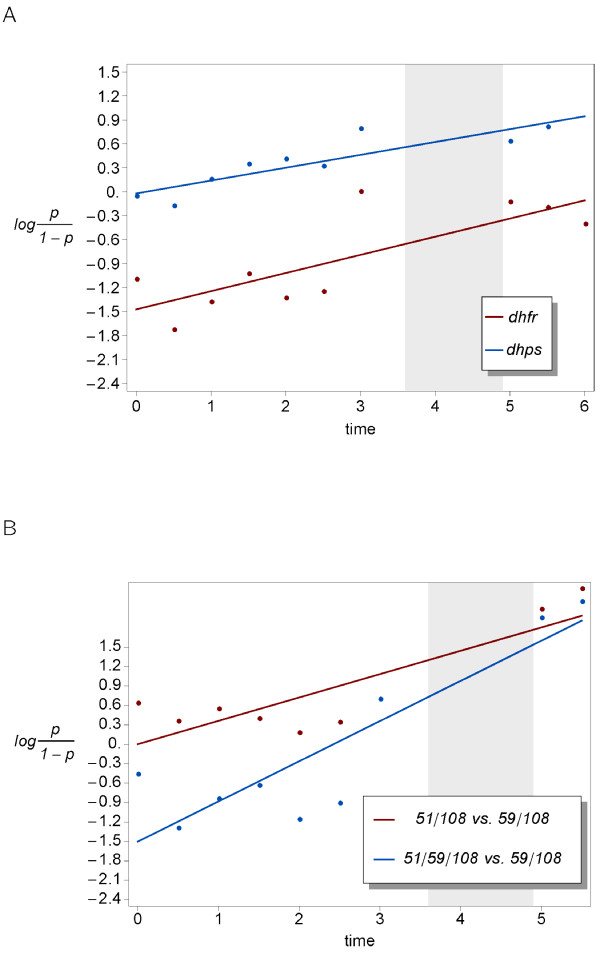
**Estimates for the strength of selection**. (**A**) 437 G/540E *vs *wildtype at *dhps *and 51I/59R/108 N *vs *51I/108 N at *dhfr*. (**B**) 51I/108 N *vs *59R/108 N and 51I/59R/108 N *vs *59R/108 N at *dhfr*.

Using linear regression, the 51I/59R/108 N allele has a selective advantage over both double mutant alleles at *dhfr*: *s_1 _= 0.013 (± 0.004) *over the 51I/108 N allele (Figure 2A), and *s_2 _= 0.036 (± 0.007) *over the 59R/108 N allele (Figure 2B). Note that s_2 _is a lower bound for the selective advantage of the 51I/59R/108 N allele over the (extinct) wildtype. Furthermore, the 51I/108 N allele has a selective advantage of *s_3 _= 0.021 (± 0.005) *over 59R/108 N allele (Figure 2B). These estimates are consistent since 1 + s_3_≈(1 + s_2_)*(1 + s_1_). At *dhps*, the 437 G/540E allele has a selective advantage s = 0.009 (± 0.002) over wildtype alleles (Figure 2A).

### Variation around genes under selection

The pattern of genetic variation linked to *dhfr *and *dhps *was examined. Number of alleles per locus (*A*) and heterozygosity (*H_e_*) was calculated as a measure of variation at each microsatellite locus (Additional file 6: Table S2).

**Figure 3:**
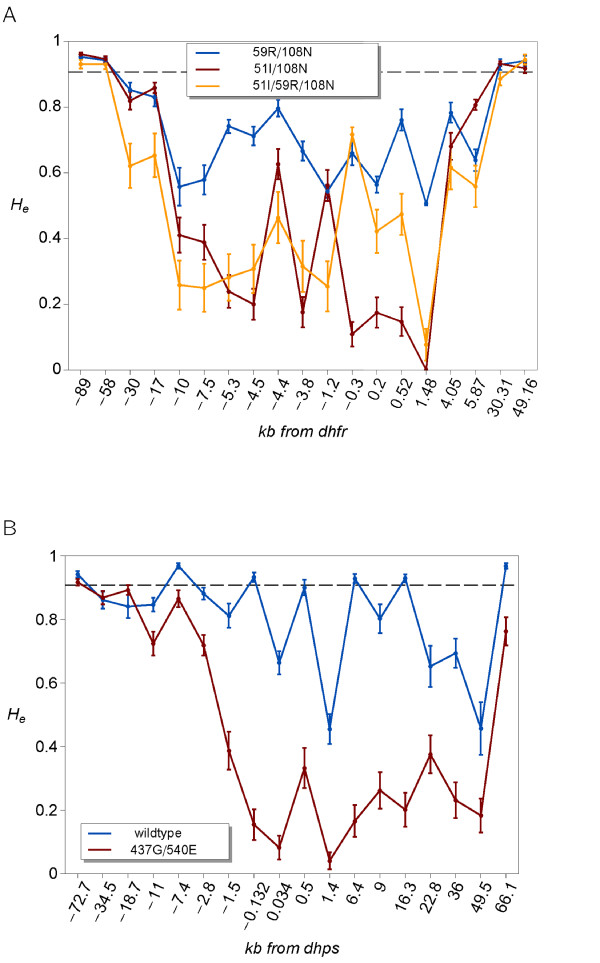
**A) Observed *H_e _*at ms loci around *dhfr *alleles 51I/59R/108 N, 51I/108 N, and 59R/108 N**. B). Observed *H_e _*around *dhps *wildtype and 437 G/540E alleles. Here, *H_e _*is calculated from single infections only. Loci are named according to their positions relative to *dhfr *or *dhps *(kb from the gene). The sampling variance is indicated by error bars. Dashed horizontal line indicates the average *H_e _*from chromosomes 2 and 3.

The number of alleles found at each microsatellite locus around *dhfr *and *dhps *alleles is shown in Additional file 7: Figure S5. There is a stronger reduction in *H_e _*surrounding *dhfr *51I/59R/108 N and 51I/108 N alleles, than the 59R/108 N allele (Figure 3A). A significant reduction in *H_e _*is observed around *dhps *437 G/540E mutant allele compared to the wildtype allele (Figure 3B).

The observed *H_e _*among *dhfr *51I/59R/108 N alleles and *dhps *437 G/540E alleles was compared to the *H_e _*predicted by a standard selective sweep model [33], using the estimates for selection coefficients. Similar models have been used elsewhere [9,21]; however, these assume that the beneficial allele has reached a frequency of nearly 100%. As an estimate for the strength of selection, s = 0.036 was used, which is a lower estimate for selective advantage of the triple mutant over the wildtype. The initial *H_e _*for *dhfr *could not be estimated because sensitive wildtype alleles were present only at marginal frequencies. Therefore, the initial *H_e _*was set to *H_e _*among infections without the triple mutant (including infections with the mixed codons N51I/S108N or C59R/S108N. The prediction underestimates *H_e _*for parasites carrying the 51I/59R/108 N triple mutant at *dhfr *(Additional file 8: Figure S6). This can be due to the assumption of 17.3 transmissions per year, which leads likely to underestimates of selection.

At *dhps *the pattern of observed *H_e _*is in general agreement with the predicted heterozygosity (Additional file 6: Table S2). *H_e _*3' to the gene is much higher than the actual observation; however, this is typical for selective sweeps that the valley of reduced heterozygosity is much more pronounced on one side of the target of selection [34].

The pattern of linkage disequilibrium (LD) in the chromosomal region surrounding *dhfr *and *dhps *is another measure of strong selection in the population (Additional file 9: Figure S7). In addition, LD between the codons involved in drug resistance of *dhfr *and *dhps *was examined. There was significant LD between codons 437 and 540 of *dhps *(Additional file 9: Figure S7).

## Discussion

### Allele populations: genetic differentiation and divergence

Western Kenya is an area of intense transmission with an entomological inoculation rate (EIR) of approximately 300 infective bites per person per year during the early 1990s, before the introduction of bed nets [25]. Therefore, there will be many multiple 'strain' infections (as many as 70% of the samples collected in this study as measured by the neutral microsatellite markers) and, as a consequence, a great amount of meiotic recombination. The large amount of recombination along with historically older parasite populations in Africa contributes to a large amount of variation in term of the number of different genetic lineages circulating in the population. Consequently, reconstructing a pattern of descent is difficult to do with absolute precision. Nevertheless, the data support an overall pattern of genetic differentiation between the major mutant alleles of *dhfr *and the *dhps *double mutant allele from any wildtype alleles in the population. Genetic differentiation between *dhfr *alleles was maintained over time. The data suggest that the 51I/108 N and 51I/59R/108 N alleles have remained distinct in the population. This can be explained by stronger selection for the 51I/59R/108 N, and the fact that the majority of these alleles were imported from Southeast Asia along a haplotype, which was not present in Kenya. Similarly, genetic differentiation between *dhfr *alleles in a Cameroonian population was previously noted [14].

The most predominant *dhfr *triple mutant haplotype in both sample sets (Kenya and Cameroon) was one previously described in Southeast Asia and has since been documented in multiple sites across Africa, including eastern Kenya [9,10,12,13,15]. This Southeast Asian haplotype appears to have diverged over time in the western Kenyan population; this result is expected for an allele that has been in a population for many years. The highly resistant Southeast Asian lineage is the most prevalent triple mutant *dhfr *lineage in both the 2002-2004 and 1992-1999 sample sets, which is consistent with studies suggesting that the Southeast Asian haplotype is the only one present in African populations [11-13]. Furthermore, a study from eastern Kenya showed only the Southeast Asian lineage for all triple mutant alleles, and this lineage was present as early as 1988 [13]. Here, this lineage is documented in western Kenya as early as 1992. The mechanisms that allowed the minor and major variant triple mutant alleles to emerge and be maintained over time cannot be completely characterized at this time since there is a paucity of serial samples from the time period prior to the widespread use of SP. Theoretically, it is possible that the triple mutant was created by mutation from a rare haplotype, which is similar to the Asian haplotype but too infrequent to be detected in the sample, and spread to high frequency; however, this alternative is highly unlikely.

The double mutant *dhfr *and *dhps *alleles are also illuminating. There is a larger number of haplotypes for these alleles than has been documented at other sites in Africa [10,13]. There are, however, fewer haplotypes for the triple mutant *dhfr *allele than for the double mutants and fewer haplotypes for the mutant *dhps *allele than the wildtype allele: results which are consistent with the hypotheses that selection is stronger on the alleles with fewer haplotypes or that these alleles are newer in the population [14,19]. Moreover, more haplotypes were found for the 59R/108 N allele than the 51I/108 N allele, and this is consistent with results that selection on the latter allele is stronger. Namely, for the 51I/108 N mutant (i) recombination breaks down the initial association with the ancestral haplotype less efficiently, and (ii) the time window in which recurrent mutations can occur and rise to detectible frequency is narrower.

### Hitchhiking and linkage disequilibrium

The shape of the curve of variation around genes under strong selection is affected by a number of factors including the strength of selection, time since the initial selective event, and the amount of recombination [32,35]. This asymmetry is consistent with hitchhiking models even when the rates of recombination and mutation are constant [34]. The asymmetry seen in the lack of variation surrounding *dhfr *and *dhps *has been documented previously for *dhfr *[19,21]. The peaks in *H_e _*around the *dhfr *triple mutant compared with the 51I/108 N double mutant at some microsatellite loci are consistent with the evidence that the triple mutant was imported from Southeast Asia. Namely, imported microsatellite alleles (which were not existing in Kenya) could have hitchhiked with the triple mutant, resulting in relatively large *H_e_*.

If resistant alleles confer a selective advantage, one can hypothesize that the genetic variation around these alleles would be substantially reduced as a result of stronger selection compared to the wildtype or less resistant/tolerant alleles in the population. This has been demonstrated in African *P. falciparum *populations previously for *dhfr *[19] and *dhps *[17]. The population of *dhfr *and *dhps *alleles in western Kenya shows dramatic levels of LD around both genes compared to neutral markers. This is expected under conditions of very strong natural selection, such as those that might occur in environments that promote the rise of drug resistance. Theoretical evidence supports the hypothesis that LD will decay rapidly after a hitchhiking event [36,37]. Here, there is LD for approximately 20 kb around both *dhfr *and *dhps*. A more advanced theoretical model is needed to evaluate the patterns seen here in western Kenya.

The data here do not demonstrate significant LD between mutant *dhfr *and *dhps *alleles - another line of evidence suggesting that selection has not occurred on any two mutant alleles at a given point in time in the population; i.e. selection on *dhfr *alleles is likely independent of that on *dhps *alleles. This finding suggests that, in a holoendemic area with high recombination, a multidrug-resistant haplotype is less likely to be maintained in the absence of drug pressure, as has been the case in areas with low transmission [20]. Additionally, since SP was a second-line treatment, joined selection pressure on *dhfr *and *dhps *must have been weaker (in the overall population) than as if it was the first-line treatment. Again, this increases the chance of meiotic recombination between *dhfr *and *dhps*.

### Selective sweeps

Sulphadoxine-pyrimethamine resistance in the Asembo Bay area was reported in the literature as early as 1982; therefore, the high proportion of mutant *dhfr *and *dhps *alleles in the population is not surprising [23]. Since the data used in this study provides longitudinal information, it was possible to directly estimate the selective parameters from the frequency changes in the various mutations. Notably, a theoretical model [38,39] tailored to *P. falciparum *justifies this approach. Thus, the results presented here are the first estimates of selective parameters from molecular data in Africa and the first for *dhps*. The results for *dhps*, in contrast to *dhfr*, revealed a greater proportion of wildtype alleles in the population. Similar data have been noted in previous studies, and Nzila *et al. *[40] have suggested that after the triple mutant *dhfr *alleles spread sufficiently through a population, *dhps *mutant alleles increase in frequency due to selection by sulphadoxine [14,40,41]. While *dhfr *double mutants were predominantly present, triple mutants increased in frequency from 1992-1999, in accordance with positive selection for those mutants. Sulphadoxine-sensitive wildtype *dhps *alleles were still present at appreciable frequency, while resistant mutant alleles increased in frequency during this observed time period.

Further evidence for strong selection for the double mutant allele is the loss of variation around *dhps *437 G/540E allele as well as high levels of LD around *dhps*. It appears as though this study has captured the *dhps *437 G/540E alleles in the middle of a selective sweep, i.e. while its frequency increases in the population. The data suggest that this population in western Kenya possibly experienced dramatically strong selection events leading to a rapid increase in frequency of resistant *dhfr *alleles, but such events took place before drug selection allowed for the rise in frequency of *dhps *resistant alleles. Indeed, this hypothesis is consistent with previous studies [1,17,42]. It would be interesting to assay this population again based on more recent samples to observe how shape and depth of the heterozygosity as well as the frequency spectrum of resistant mutations have changed as a result of a change in drug policy. In particular, by employing the regression approach presented here, it would be possible to estimate the amount of metabolic costs associated with drug-resistance of various *dhfr *and *dhps *mutations in natural settings.

Soft selective sweeps, as opposed to the traditional "hard" selective sweep proposed by Maynard Smith and Haigh [32], are a case where multiple alleles are favoured by selection and, consequently, multiple genetic backgrounds hitchhike with the alleles under selection [43,44]. The result will be an increase in the amount of variation surrounding the selected allele as compared to a hard selective sweep. The presence of different predominant haplotypes for *dhfr *alleles together with reduced variation along the chromosome for each of these is evidence of selection on each of these alleles - a soft selective sweep.

Typically, the classical "hard" sweep of the Southeast Asian triple mutant allele has been suggested for *dhfr *alleles. These arguments, however, do not describe the dynamics of drug resistant mutations for *Plasmodium*, especially in Africa. This study has led to the hypothesis that soft sweeps involving drug resistant alleles should be more common in Africa since drug pressure is effectively lower. Indeed, there is a higher proportion of asymptomatic (untreated) infections due to higher levels of acquired immunity. It is worth noting that the data from [10] is suggestive of a soft sweep in South African and Tanzanian populations. Nevertheless, understanding the factors leading to soft sweeps for pyrimethamine resistance, and any other form of drug resistance that involves such a complex pattern of alleles, requires more studies.

Multiple origins and soft sweeps are expected to be common if mutation rates are high or population sizes are large; specifically if the population-based mutation parameter *2N_e_μ > 0.01 *[43]. The mutation rate for pyrimethamine resistance has been estimated to be *10^-9 ^*[45] and the estimated population size for African *P. falciparum *populations based on mtDNA sequence variation is about *10^5 ^*[46,47]. Given these estimates (*2N_e_μ = 2 × 10^-4^*), a first approximation is that soft sweeps are expected to be rare [43]. Note that estimates for N_e _may have limited meaning, since these estimates reflect the fact that some characteristic is equivalent to a Wright-Fisher model with sample size N_e_. The complexity of the *P. falciparum *transmission cycle implies that there are processes that exceed the simplicity of standard population genetic assumptions [38]. However, the population-genetic analyses performed in this article are justified by the theoretical results of Schneider and Kim [38,39]. Summarizing, soft sweeps might be more common than naively expected.

### Strength of selection

In [9], a similar approach as here was used to estimate the selective strength at *dhfr *in a Southeast Asian population; however, there are crucial differences. The strength of selection was estimated from genetic data, whereas the prior study used historic data from clinical treatment failures and clinical success. The latter is problematic, because clinical failures do not directly correlate with the presence of resistant mutations and there could be multiple variables affecting a patient's treatment outcome. Moreover, the data are acquired from symptomatic infections (asymptomatic infections do not need treatment), which implies an overestimation of the strength of selection since it masks the disadvantage of resistance due to metabolic costs that are only apparent in untreated infections. Notably, the use of longitudinal data allowed for a comprehensive analysis on the strength of selection acting on specific mutants rather than an overall average of selection. Nair *et al. *[9] assumed six transmission cycles per year and an inbreeding adjustment factor of 80%; parameters that properly describe an area with lower transmission and lower frequency of multiple infections but do not properly describe holo-endemic areas such as western Kenya. In this case, 17.3 transmission cycles per year were used, which, assuming an incubation period of three weeks corresponds to infections over the entire course of a year. The choice of 21 days was based on the extremely high transmission rates, the fact that a mosquito becomes infective approximately 10 days after the blood meal, and that it will take seven to 11 days until gametocytaemia peaks in infected patients. Also the inbreeding was adjusted to 40%, which is in agreement with the percentage of observed multiple infections (63%-70%). Whereas the absolute values of s depend on these assumptions, the relative pattern observed does not. Nevertheless, it is straightforward to re-calculate *s *assuming different numbers of transmission cycles per year (see methods). It is worth nothing that there is not such a thing as a universal "*s*"; thus, conclusions should be made based on general patterns and relative differences at a local level.

For the purpose of estimating the selective strengths, sample sizes were rather small due to multiple infections and the inability to discern alleles; however, an approach was pursued that included as many isolates as possible. Regardless of its limitations, the results clearly indicate that drug selection due to a combination drug therapy operates differently at each individual locus, and for individual alleles per locus. Overall, the study provides direct temporal evidence of differential selection acting on *dhfr *and *dhps *mutations associated with resistance in Africa.

## Conclusions

The three signatures of a selective sweep in a population: altered distribution of polymorphic sites along the chromosome, altered allele frequency spectrum, and an increase in the amount of linkage disequilibrium, are all seen in *dhfr *and *dhps *allele populations in western Kenya. The independent origination, genetic differentiation, and maintenance of alleles allude to the fact that rapid, dynamic events in the clinical and ecological settings have given rise to the patterns of resistant mutations we see today. Regardless of the fact that SP is a combination drug therapy, the strength of selection on the two loci is different and the drug by itself does not appear to select for "multidrug"-resistant parasites in areas with high recombination rate. The various estimates for the selective strengths on various mutant alleles, allow for a more complete understanding of the evolutionary dynamics associated with drug-resistance. Thus, the local demographic history (effective population size and recombination rate) needs to be taken into account when investigating the rise of multi-resistant genotypes in *Plasmodium *populations.

## Competing interests

The authors declare that they have no competing interests.

## Authors' contributions

AMM, KAS, AAE, and VU designed the study and drafted the manuscript. AMM, SMG, and ZZ carried out the molecular genetics studies. KAS carried out the theoretical and statistical analyses. SK, FK, YPS, LS, and AAL participated in the design and coordination of sample collection. All authors read and approved the final manuscript.

## Supplementary Material

Additional file 1 **Table S1**. PCR primers used for dhps microsatellite amplification. Click here for file

Additional file 2 **Figure S1**. Haplotype frequencies for dhfr alleles: A) 59R/108N (*n = 40*), B) 51I/108N (*n = 72*), and C) 51I/59R/108N (*n = 26*). Haplotypes are along the X axis and frequency in the sample set is along the y axis. Click here for file

Additional file 3 **Figure S2**. Haplotype frequencies for dhps alleles: A) wildtype (*n = 57*) and B) 437G/540E (*n = 95*). Haplotypes are along the X axis and frequency in the sample set is along the y axis. Click here for file

Additional file 4 **Figure S3**. Relationships among 95 8-locus dhfr microsatellite haplotypes from populations in Western Kenya as determined by eBURST analysis. Samples from 1992-1999 (*n = 134 *samples) and 2002-2004 (*n = 37 *samples) were used. Each line connects haplotypes that are identical at 7 of 8 loci. The size of the circles is proportional to the number of isolates of the given haplotype. The blue circles represent founders for the clusters and the yellow circle represent subgroup founders. Black circles without any shading represent haplotypes only present for the samples collected in 1992-1999, green shading represents haplotypes present only in the 2002-2004 collection, and pink shading represents haplotypes present in both collections. 51I/59R/108N haplotypes circled in red are triple mutants that originated independently from the SE Asian haplotype. Two genotypes that include the mutation 164L are noted not being connected to any other haplotype. Click here for file

Additional file 5 **Figure S4**. Relationships among 128 9-locus dhps microsatellite haplotypes from western Kenya as determined by eBURST analysis. Each line connects haplotypes that are identical at 8 out of 9 loci. The size of the circles is proportional to the number of isolates of the given haplotype. Haplotypes shown as single points differ from the other haplotypes by alleles in at least 2 loci. The central complex represents haplotypes from the 437G/540E allele. A total of 44 samples with the wildtype allele and 84 samples with the 437G/540E allele were utilized for this analysis. Click here for file

Additional file 6 **Table S2**. Number of alleles (A) and heterozygosity (*H_e_*) per locus and averaged over loci. Click here for file

Additional file 7 **Figure S5**. Relationships among 128 9-locus *dhps *microsatellite haplotypes from western Kenya as determined by eBURST analysis. Each line connects haplotypes that are identical at 8 out of 9 loci. The size of the circles is proportional to the number of isolates of the given haplotype. Haplotypes shown as single points differ from the other haplotypes by alleles in at least 2 loci. The central complex represents haplotypes from the 437G/540E allele. A total of 44 samples with the wildtype allele and 84 samples with the 437G/540E allele were utilized for this analysis. Click here for file

Additional file 8 **Figure S6**. Observed and predicted *H_e _*at ms loci around the (A) dhfr allele 51I/59R/108N and (B) *dhps *allele 437G/540E. For the dhfr prediction (A) we used *H_e _*among all samples that did not include the 51I/59R/108N triple mutant (i.e. infections with the mixed codons N51I/S108N and C59R/S108N were included). For the *dhps *prediction (B) we used *H_e _*among wildtype alleles as an estimate for the initial heterozygosity. Loci are labelled according to their positions relative to *dhfr *or *dhps *(kb from the gene). Sampling variance is indicated by error bars. Click here for file

Additional file 9 **Figure S7**. Pairwise LD between microsatellite loci on different chromosomes (A) and between sites in *dhfr *and *dhps *(B). Each cell represents one comparison between polymorphic pairs of loci. Gray cells represent significance at p value < 0.01. (A) The position of *dhfr *and *dhps *along the chromosome is denoted by the gray bar. The location of each microsatellite locus is at the top of the matrix (loci are named according to their positions relative to *dhfr *or *dhps *or along chromosome 2 or 3 according to the 3D7 genome sequence available from NCBI). (B) Pairwise LD between sites in *dhfr *(51, 59, 108) and *dhps *(436, 437, 540). Click here for file

## References

[B1] Gregson A Plowe CV Mechanisms of resistance of malaria parasites to antifolates Pharmacol Rev 2005 57 117 145 10.1124/pr.57.1.4 15734729

[B2] Talisuna AO Bloland P D'Alessandro U History, dynamics, and public health importance of malaria parasite resistance Clin Microbiol Rev 2004 17 235 254 10.1128/CMR.17.1.235-254.2004 14726463 PMC321461

[B3] Dondorp AM Nosten F Yi P Das D Phyo AP Tarning J Lwin KM Ariey F Hanpithakpong W Lee SJ Ringwald P Silamut K Imwong M Chotinavich K Lim P Herdman T An SS Yeung S Singhasivanon P Day NP Lindegardh N Socheat D White NJ Artemisinin resistance in *Plasmodium falciparum *malaria N Engl J Med 2009 361 455 467 10.1056/NEJMoa0808859 19641202 PMC3495232

[B4] Dondorp AM Yeung S White L Nguon C Day NP Socheat D Von-Seidlein L Artemisinin resistance: current status and scenarios for containment Nat Rev Microbiol 2010 8 272 280 20208550 10.1038/nrmicro2331

[B5] Hayton K Su XZ Genetic and biochemical aspects of drug resistance in malaria parasites Curr Drug Targets Infect Disord 2004 4 1 10 15032630 10.2174/1568005043480925

[B6] Cortese JF Plowe CV Antifolate resistance due to new and known *Plasmodium falciparu *dihydrofolate reductase mutations expressed in yeast Mol Biochem Parasitol 1998 94 205 214 10.1016/S0166-6851(98)00075-9 9747971

[B7] Plowe CV Kublin JG Doumbo O *P. falciparum *dihydrofolate reductase and dihydropteroate synthase mutations: epidemiology and role in clinical resistance to antifolates Drug Resist Updat 1998 1 389 396 10.1016/S1368-7646(98)80014-9 17092820

[B8] Triglia T Wang P Sims PF Hyde JE Cowman AF Allelic exchange at the endogenous genomic locus in *Plasmodium falciparum *proves the role of dihydropteroate synthase in sulfadoxine-resistant malaria EMBO J 1998 17 3807 3815 10.1093/emboj/17.14.3807 9669998 PMC1170716

[B9] Nair S Williams JT Brockman A Paiphun L Mayxay M Newton PN Guthmann JP Smithuis FM Hien TT White NJ Nosten F Anderson TJ A selective sweep driven by pyrimethamine treatment in Southeast Asian malaria parasites Mol Biol Evol 2003 20 1526 1536 10.1093/molbev/msg162 12832643

[B10] Roper C Pearce R Bredenkamp B Gumede J Drakeley C Mosha F Chandramohan D Sharp B Antifolate antimalarial resistance in southeast Africa: a population-based analysis Lancet 2003 361 1174 1181 10.1016/S0140-6736(03)12951-0 12686039

[B11] Roper C Pearce R Nair S Sharp B Nosten F Anderson T Intercontinental spread of pyrimethamine-resistant malaria Science 2004 305 1124 10.1126/science.1098876 15326348

[B12] Maiga O Djimde AA Hubert V Renard E Aubouy A Kironde F Nsimba B Koram K Doumbo OK Le-Bras J Clain J A shared Asian origin of the triple-mutant dhfr allele in *Plasmodium falciparum *from sites across Africa J Infect Dis 2007 196 165 172 10.1086/518512 17538897

[B13] Certain LK Briceno M Kiara SM Nzila AM Watkins WM Sibley CH Characteristics of Plasmodium falciparum dhfr haplotypes that confer pyrimethamine resistance, Kilifi, Kenya, 1987-2006 J Infect Dis 2008 197 1743 1751 10.1086/588198 18513156 PMC2680814

[B14] McCollum AM Basco LK Tahar R Udhayakumar V Escalante AA Hitchhiking and selective sweeps of *Plasmodium falciparum *sulfadoxine and pyrimethamine resistance alleles in a population from central Africa Antimicrob Agents Chemother 2008 52 4089 4097 10.1128/AAC.00623-08 18765692 PMC2573158

[B15] McCollum AM Poe AC Hamel M Huber C Zhou Z Shi YP Ouma P Vulule J Bloland P Slutsker L Barnwell JW Udhayakumar V Escalante AA Antifolate resistance in *Plasmodium falciparum*: multiple origins and identification of novel dhfr alleles J Infect Dis 2006 194 189 197 10.1086/504687 16779725

[B16] Alam MT Vinayak S Congpuong K Wongsrichanalai C Satimai W Slutsker L Escalante AA Barnwell JW Udhayakumar V Tracking origins and spread of sulfadoxine-resistant *Plasmodium falciparum *dhps alleles in Thailand Antimicrob Agents Chemother 2011 55 155 164 10.1128/AAC.00691-10 20956597 PMC3019650

[B17] Pearce RJ Pota H Evehe MS Ba-el H Mombo-Ngoma G Malisa AL Ord R Inojosa W Matondo A Diallo DA Mbacham W Van den-Broek IV Swarthout TD Getachew A Dejene S Grobusch MP Njie F Dunyo S Kweku M Owusu-Agyei S Chandramohan D Bonnet M Guthmann JP Clarke S Barnes KI Streat E Katokele ST Uusiku P Agboghoroma CO Elegba OY Multiple origins and regional dispersal of resistant dhps in African *Plasmodium falciparum *malaria PLoS Med 2009 6 e1000055 10.1371/journal.pmed.1000055 19365539 PMC2661256

[B18] Vinayak S Alam MT Mixson-Hayden T McCollum AM Sem R Shah NK Lim P Muth S Rogers WO Fandeur T Barnwell JW Escalante AA Wongsrichanalai C Ariey F Meshnick SR Udhayakumar K Origin and evolution of sulfadoxine resistant *Plasmodium falciparum* PLoS Pathog 2010 6 e1000830 10.1371/journal.ppat.1000830 20360965 PMC2847944

[B19] Pearce R Malisa A Kachur SP Barnes K Sharp B Roper C Reduced variation around drug-resistant *dhf *alleles in African *Plasmodium falciparum* Mol Biol Evol 2005 22 1834 1844 10.1093/molbev/msi177 15917494

[B20] McCollum AM Mueller K Villegas L Udhayakumar V Escalante AA Common origin and fixation of *Plasmodium falciparum dhf *and *dhp *mutations associated with sulfadoxine-pyrimethamine resistance in a low-transmission area in South America Antimicrob Agents Chemother 2007 51 2085 2091 10.1128/AAC.01228-06 17283199 PMC1891388

[B21] Nash D Nair S Mayxay M Newton PN Guthmann JP Nosten F Anderson TJ Selection strength and hitchhiking around two anti-malarial resistance genes Proc Biol Sci 2005 272 1153 1161 10.1098/rspb.2004.3026 16024377 PMC1559806

[B22] Terlouw DJ Nahlen BL Courval JM Kariuki SK Rosenberg OS Oloo AJ Kolczak MS Hawley WA Lal AA Kuile FO Sulfadoxine-pyrimethamine in treatment of malaria in Western Kenya: increasing resistance and underdosing Antimicrob Agents Chemother 2003 47 2929 2932 10.1128/AAC.47.9.2929-2932.2003 12936996 PMC182608

[B23] Nguyen-Dinh P Spencer HC Chemangey-Masaba S Churchill FC Susceptibility of *Plasmodium falciparum *to pyrimethamine and sulfadoxine/pyrimethamine in Kisumu, Kenya Lancet 1982 1 823 825 6122056 10.1016/s0140-6736(82)91873-6

[B24] Bloland PB Boriga DA Ruebush TK McCormick JB Roberts JM Oloo AJ Hawley W Lal A Nahlen B Campbell CC Longitudinal cohort study of the epidemiology of malaria infections in an area of intense malaria transmission II. Descriptive epidemiology of malaria infection and disease among children Am J Trop Med Hyg 1999 60 641 648 10348241 10.4269/ajtmh.1999.60.641

[B25] Beier JC Perkins PV Onyango FK Gargan TP Oster CN Whitmire RE Koech DK Roberts CR Characterization of malaria transmission by Anopheles (Diptera: Culicidae) in western Kenya in preparation for malaria vaccine trials J Med Entomol 1990 27 570 577 2388233 10.1093/jmedent/27.4.570

[B26] Zhou Z Poe AC Limor J Grady KK Goldman I McCollum AM Escalante AA Barnwell JW Udhayakumar V Pyrosequencing, a high-throughput method for detecting single nucleotide polymorphisms in the dihydrofolate reductase and dihydropteroate synthetase genes of *Plasmodium falciparum* J Clin Microbiol 2006 44 3900 3910 10.1128/JCM.01209-06 16957045 PMC1698350

[B27] Feil EJ Li BC Aanensen DM Hanage WP Spratt BG eBURST: inferring patterns of evolutionary descent among clusters of related bacterial genotypes from multilocus sequence typing data J Bacteriol 2004 186 1518 1530 10.1128/JB.186.5.1518-1530.2004 14973027 PMC344416

[B28] Wright S The interpretation of population structure by F-statistics with special regards to systems of mating Evolution 1965 19 395 420 10.2307/2406450

[B29] Excoffier L Laval G Schneider S Arlequin ver. 3.0: an integrated software package for population genetics data analysis Evolutionary Bioinformatics Online 2005 1 47 50 19325852 PMC2658868

[B30] Park SDE Trypanotolerance in West African cattle and the population genetic effects of selection PhD thesis, University of Dublin 2001

[B31] Raymond M Rousset F An exact test for population differentiation Evolution 1995 49 1280 1283 10.2307/2410454 28568523

[B32] Maynard Smith J Haigh J The hitch-hiking effect of a favourable gene Genet Res 1974 23 23 35 10.1017/S0016672300014634 4407212

[B33] Wiehe T The effect of selective sweeps on the variance of the allele distribution of a linked multiallele locus: hitchhiking of microsatellites Theor Popul Biol 1998 53 272 283 10.1006/tpbi.1997.1346 9679322

[B34] Kim Y Stephan W Detecting a local signature of genetic hitchhiking along a recombining chromosome Genetics 2002 160 765 777 11861577 10.1093/genetics/160.2.765 PMC1461968

[B35] Kaplan NL Hudson RR Langley CH The "hitchhiking effect" revisited Genetics 1989 123 887 899 2612899 10.1093/genetics/123.4.887 PMC1203897

[B36] McVean G The structure of linkage disequilibrium around a selective sweep Genetics 2007 175 1395 1406 17194788 10.1534/genetics.106.062828 PMC1840056

[B37] Stephan W Song YS Langley CH The hitchhiking effect on linkage disequilibrium between linked neutral loci Genetics 2006 172 2647 2663 16452153 10.1534/genetics.105.050179 PMC1456384

[B38] Schneider KA Kim Y An analytical model for genetic hitchhiking in the evolution of antimalarial drug resistance Theor Popul Biol 2010 78 93 108 10.1016/j.tpb.2010.06.005 20600206 PMC2916054

[B39] Schneider KA Kim Y Approximations for the hitchhiking effect caused by the evolution of antimalarial-drug resistance J Math Biol 2011 62 789 832 10.1007/s00285-010-0353-9 20623287 PMC3242009

[B40] Nzila AM Mberu EK Sulo J Dayo H Winstanley PA Sibley CH Watkins WM Towards an understanding of the mechanism of pyrimethamine-sulfadoxine resistance in *Plasmodium falciparum*: genotyping of dihydrofolate reductase and dihydropteroate synthase of Kenyan parasites Antimicrob Agents Chemother 2000 44 991 996 10.1128/AAC.44.4.991-996.2000 10722502 PMC89803

[B41] Hapuarachchi HC Dayanath MY Bandara KB Abeysundara S Abeyewickreme W De-Silva NR Hunt SY Sibley CH Point mutations in the dihydrofolate reductase and dihydropteroate synthase genes of *Plasmodium falciparum *and resistance to sulfadoxine-pyrimethamine in Sri Lanka Am J Trop Med Hyg 2006 74 198 204 16474070

[B42] Nzila A Ochong E Nduati E Gilbert K Winstanley P Ward S Marsh K Why has the dihydrofolate reductase 164 mutation not consistently been found in Africa yet? Trans R Soc Trop Med Hyg 2005 99 341 346 10.1016/j.trstmh.2004.07.002 15780340

[B43] Pennings PS Hermisson J Soft sweeps II-molecular population genetics of adaptation from recurrent mutation or migration Mol Biol Evol 2006 23 1076 1084 10.1093/molbev/msj117 16520336

[B44] Pennings PS Hermisson J Soft sweeps III: the signature of positive selection from recurrent mutation PLoS Genet 2006 2 e186 10.1371/journal.pgen.0020186 17173482 PMC1698945

[B45] Paget-McNicol S Saul A Mutation rates in the dihydrofolate reductase gene of *Plasmodium falciparum* Parasitology 2001 122 497 505 11393822 10.1017/s0031182001007739

[B46] Joy DA Feng X Mu J Furuya T Chotivanich K Krettli AU Ho M Wang A White NJ Suh E Beerli P Su XZ Early origin and recent expansion of *Plasmodium falciparum* Science 2003 300 318 321 10.1126/science.1081449 12690197

[B47] White NJ Pongtavornpinyo W The de novo selection of drug-resistant malaria parasites Proc Biol Sci 2003 270 545 554 10.1098/rspb.2002.2241 12641911 PMC1691263

