# Supplementary material for: Differences in selective pressure on dhps and dhfr drug resistant mutations in western Kenya
Source: Malar J. 2012 Mar 22;11:77. doi: 10.1186/1475-2875-11-77 (PMC3338400; doi:10.1186/1475-2875-11-77)
Supplement: Additional file 1 — Table S1. PCR primers used for dhps microsatellite amplification. [file 1475-2875-11-77-S1.DOC]

**Table 1S.** PCR primers used for *dhps* microsatellite amplification.

| **Locus^a^** | **Previously published locus name^b^** | **Sequence (5’→3’) - direction** |
| --- | --- | --- |
| -72.7 kb *dhps* | 71.6 kb *dhps* | CAAATGCACATGAGTGAGT |
|  |  | ACGTGAATGACAGTGCATTTC |
| -34.5 kb *dhps* | 33.1 kb *dhps* | CCTTTTATAAGATTATTGTATCT |
|  |  | CAACTAATATTGCCACGATA |
| -18.7 kb *dhps* | 17.5 kb *dhps* | GGAGAATCAGAGAAATAC |
|  |  | TGTTGTACAAATATACG |
| -11 kb *dhps* | 9.8 kb *dhps* | TGCGGGTATAATACATTA |
|  |  | AACTTATACGTATCTAAAG |
| -7.4 kb *dhps* | 6.19 kb *dhps* | TACAGCACTTAATGTAAATGGAG |
|  |  | TTTTAACTTGTATCAAGAAAT |
| -2.8 kb *dhps* | 1.59 kb *dhps* | CACATGTAAATGCATATTTATG |
|  |  | ATGTTTGAACCCCTTAATTTA |
| -1.5 kb *dhps* | 0.144 kb *dhps* | CATAATATGAAGAGACTGAAAGTT |
|  |  | TGTCTTGAAGGACAACACATAGATG |
| -0.132 kb *dhps* | 0.06 kb *dhps* | TAGATTTCTTTACGCAAAAT |
|  |  | AAATATTTGCGCCAAACTTT |
| 0.034 kb *dhps* | -0.8 kb *dhps* | gaccaagtgtaatttac^c^ |
|  |  | AGAGTACTTGACATATAATGAGCATG^c^ |
|  |  | *(f)ggaaagtgcaaacatgt^c^ |
| 0.5 kb *dhps* | -1.6 kb *dhps* | AGGACTGATCATATTACCAAG |
|  |  | AGGAAAGTGTACGACGTTTATTGAATG |
| 1.4 kb *dhps* | -2.5 kb *dhps* | AGGAGGTTTCCCTTCACTCCATCT |
|  |  | GCATTCACACCAGTCTGCCTTCAA |
| 6.4 kb *dhps* | -7.4 kb *dhps* | CCTGAAAAGTGAGGAATGCG |
|  |  | AGCTTTTCTCCGACAAACCAAG |
| 9 kb *dhps* | -10 kb *dhps* | TGTGAAGAGAATTATCAGGAATG |
|  |  | TGGAATTCATATTAATTGTAC |
|  |  | *(f)GAATAAATTAATTACACACGGAA |
| 16.3 kb *dhps* | -17 kb *dhps* | TGAAATAGTTACACTTAATATAA |
|  |  | ACAAAAGGATATTTATAGGTATGA |
|  |  | *(r)AAAGTGTGCGCTATTGTGTTAA |
| 22.8 kb *dhps* | -23.5 kb *dhps* | TATCGACGTAGACGGATTAA |
|  |  | TTTATACCCATTTGGCATAAA |
| 36 kb *dhps* | -36.8 kb *dhps* | GATGATAATGGCGAACACAGTGCG |
|  |  | TACAAATTATGGAATCACATTTATAC |
| 49.5 kb *dhps* | -50 kb *dhps* | CCGGTGCCAATGTCAAGCCTTAT |
|  |  | ACGGAATATCAGTGGTCTTATTTGAATG |
| 66.1 kb *dhps* | -66.6 kb *dhps* | ACCTATAATCGGCACATAACGCTG |
|  |  | GTCCATATGCAATAACTACTTCTCCTTG |

Loci are named relative to their position to *dhps* on chromosome 8. Forward primers are listed first and reverse primers are listed second for each locus. * denotes primers that are used in a nested reaction followed by the orientation in parentheses. ^a^ The primer name reflecting the corrected position and orientation, ^b^ the primer name previously published (McCollum *et al* [14,15], ^c^ denotes primers originally published from Roper *et al* [11].

rwise LD between sites in *dhfr* (51, 59, 108) and *dhps* (436, 437, 540).
