# Supplementary material for: Differences in selective pressure on dhps and dhfr drug resistant mutations in western Kenya
Source: Malar J. 2012 Mar 22;11:77. doi: 10.1186/1475-2875-11-77 (PMC3338400; doi:10.1186/1475-2875-11-77)
Supplement: Additional file 2 — Figure S1. Haplotype frequencies for dhfr alleles: A) 59R/108N (n = 40), B) 51I/108N (n = 72), and C) 51I/59R/108N (n = 26). Haplotypes are along the X axis and frequency in the sample set is along the y axis. [file 1475-2875-11-77-S2.DOC]

B

C

**Figure 1S.** Haplotype frequencies for *dhfr* alleles: A) 59R/108N (n=40), B) 51I/108N (n=72), and C) 51I/59R/108N (n=26). Haplotypes are along the x axis and frequency in the sample set is along the y axis.
