# Supplementary material for: Differences in selective pressure on dhps and dhfr drug resistant mutations in western Kenya
Source: Malar J. 2012 Mar 22;11:77. doi: 10.1186/1475-2875-11-77 (PMC3338400; doi:10.1186/1475-2875-11-77)
Supplement: Additional file 3 — Figure S2. Haplotype frequencies for dhps alleles: A) wildtype (n = 57) and B) 437G/540E (n = 95). Haplotypes are along the X axis and frequency in the sample set is along the y axis. [file 1475-2875-11-77-S3.DOC]

A

B

B

**Figure 2S**. Haplotype frequencies for *dhps* alleles: A) wildtype (n=57) and B) 437G/540E (n=95). Haplotypes are along the x axis and frequency in the sample set is along the y axis.
