# Supplementary material for: Differences in selective pressure on dhps and dhfr drug resistant mutations in western Kenya
Source: Malar J. 2012 Mar 22;11:77. doi: 10.1186/1475-2875-11-77 (PMC3338400; doi:10.1186/1475-2875-11-77)
Supplement: Additional file 4 — Figure S3. Relationships among 95 8-locus dhfr microsatellite haplotypes from populations in Western Kenya as determined by eBURST analysis. Samples from 1992-1999 (n = 134 samples) and 2002-2004 (n = 37 samples) were used. Each line connects haplotypes that are identical at 7 of 8 loci. The size of the circles is proportional to the number of isolates of the given haplotype. The blue circles represent founders for the clusters and the yellow circle represent subgroup founders. Black circles without any shading represent haplotypes only present for the samples collected in 1992-1999, green shading represents haplotypes present only in the 2002-2004 collection, and pink shading represents haplotypes present in both collections. 51I/59R/108N haplotypes circled in red are triple mutants that originated independently from the SE Asian haplotype. Two genotypes that include the mutation 164L are noted not being connected to any other haplotype. [file 1475-2875-11-77-S4.DOC]

B

B


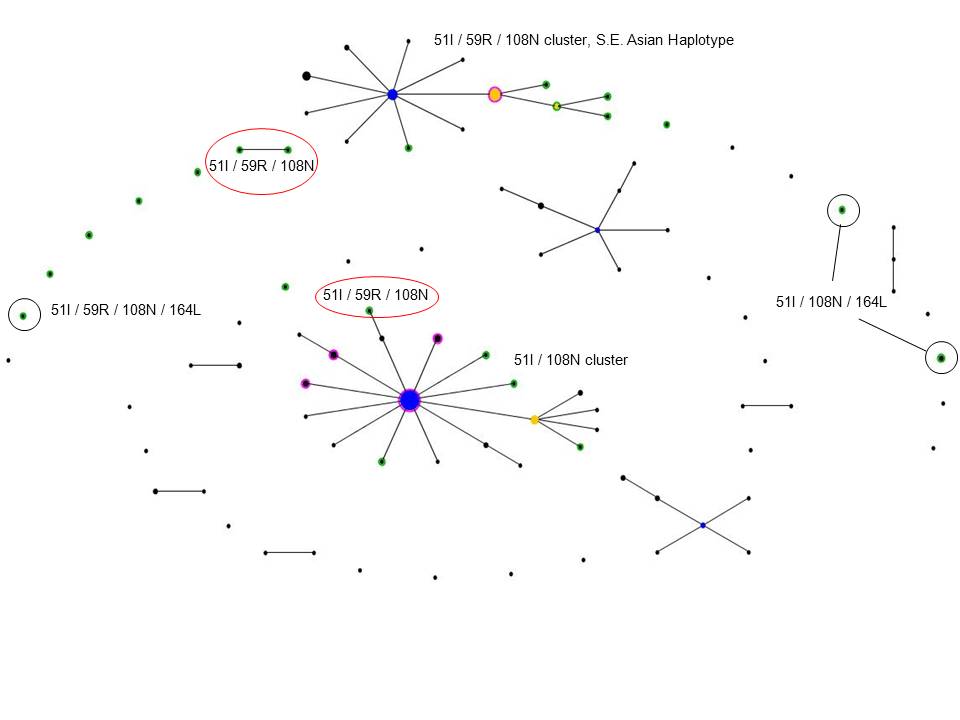


**Figure 3S.** Relationships among 95 8-locus *dhfr* microsatellite haplotypes from populations in Western Kenya as determined by eBURST analysis. Samples from 1992-1999 (n=134 samples) and 2002-2004 (n=37 samples) were used. Each line connects haplotypes that are identical at 7 of 8 loci. The size of the circles is proportional to the number of isolates of the given haplotype. The blue circles represent founders for the clusters and the yellow circle represent subgroup founders. Black circles without any shading represent haplotypes only present for the samples collected in 1992-1999, green shading represents haplotypes present only in the 2002-2004 collection, and pink shading represents haplotypes present in both collections. 51I/59R/108N haplotypes circled in red are triple mutants that originated independently from the SE Asian haplotype. Two genotypes that include the mutation 164L are noted not being connected to any other haplotype.
