# Supplementary material for: Differences in selective pressure on dhps and dhfr drug resistant mutations in western Kenya
Source: Malar J. 2012 Mar 22;11:77. doi: 10.1186/1475-2875-11-77 (PMC3338400; doi:10.1186/1475-2875-11-77)
Supplement: Additional file 5 — Figure S4. Relationships among 128 9-locus dhps microsatellite haplotypes from western Kenya as determined by eBURST analysis. Each line connects haplotypes that are identical at 8 out of 9 loci. The size of the circles is proportional to the number of isolates of the given haplotype. Haplotypes shown as single points differ from the other haplotypes by alleles in at least 2 loci. The central complex represents haplotypes from the 437G/540E allele. A total of 44 samples with the wildtype allele and 84 samples with the 437G/540E allele were utilized for this analysis. [file 1475-2875-11-77-S5.DOC]

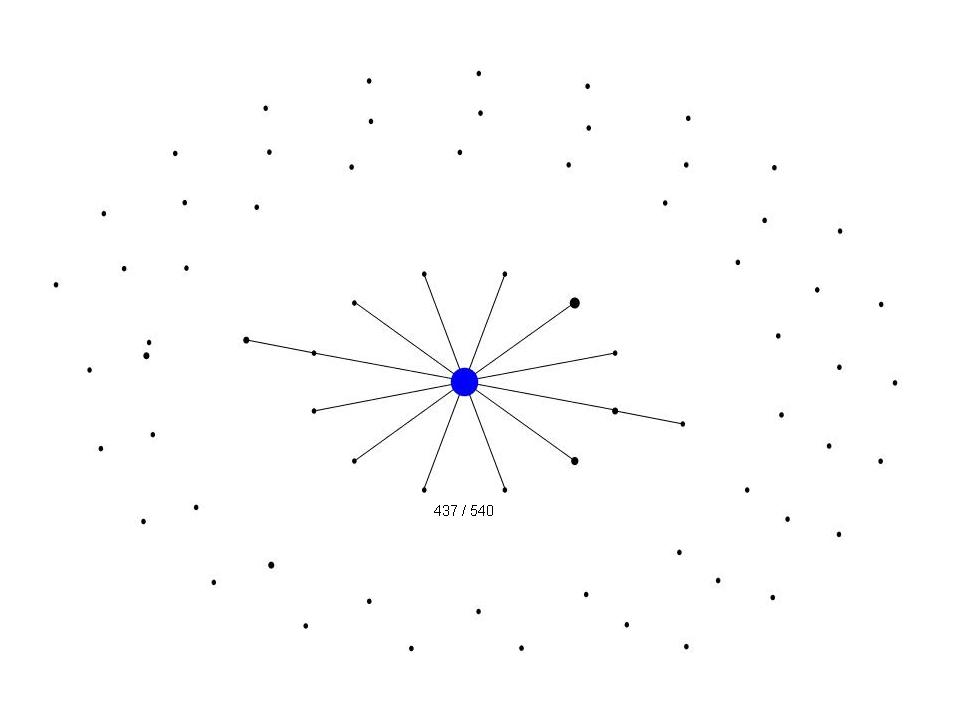


**Figure 4S.** Relationships among 128 9-locus *dhps* microsatellite haplotypes from western Kenya as determined by eBURST analysis. Each line connects haplotypes that are identical at 8 out of 9 loci. The size of the circles is proportional to the number of isolates of the given haplotype. Haplotypes shown as single points differ from the other haplotypes by alleles in at least 2 loci. The central complex represents haplotypes from the 437G / 540E allele. A total of 44 samples with the wildtype allele and 84 samples with the 437G / 540E allele were utilized for this analysis.
