# Supplementary material for: Differences in selective pressure on dhps and dhfr drug resistant mutations in western Kenya
Source: Malar J. 2012 Mar 22;11:77. doi: 10.1186/1475-2875-11-77 (PMC3338400; doi:10.1186/1475-2875-11-77)
Supplement: Additional file 6 — Table S2. Number of alleles (A) and heterozygosity (He) per locus and averaged over loci. [file 1475-2875-11-77-S6.DOC]

**Table 2S.** Number of alleles (*A*) and heterozygosity (*H_e_*) per locus and averaged over loci.

| Loci on chromosome 4 | | |  | Loci on chromosome 8 | | |  | Loci on chromosomes 2 and 3 | | |
| --- | --- | --- | --- | --- | --- | --- | --- | --- | --- | --- |
|  | *A* | *H_e_* ± SD |  |  | *A* | *H_e_* ± SD |  |  | *A* | *H_e_* ± SD |
| ch4_-89kb | 48 | 0.9609 ± 0.00 |  | ch8_-72.7kb | 41 | 0.9099 ± 0.01 |  | ch2_302kb | 21 | 0.9213 ± 0.00 |
| ch4_-58kb | 47 | 0.9453 ± 0.01 |  | ch8_-34.5kb | 15 | 0.3312 ± 0.05 |  | ch2_313kb | 39 | 0.9536 ± 0.00 |
| ch4_-30kb | 26 | 0.8470 ± 0.01 |  | ch8_-18.7kb | 12 | 0.4854 ± 0.04 |  | ch2_342kb | 27 | 0.9327 ± 0.00 |
| ch4_-17kb | 29 | 0.8896 ± 0.01 |  | ch8_-11kb | 13 | 0.7222 ± 0.02 |  | ch2_380kb | 25 | 0.9237 ± 0.00 |
| ch4_-10kb | 9 | 0.5806 ± 0.03 |  | ch8_-7.4kb | 21 | 0.7189 ± 0.03 |  | ch2_403kb | 15 | 0.8314 ± 0.01 |
| ch4_-7.5kb | 5 | 0.6072 ± 0.02 |  | ch8_-2.8kb | 17 | 0.7061 ± 0.02 |  | mean | 25.4 | 0.9125 |
| ch4_-5.3kb | 9 | 0.6480 ± 0.02 |  | ch8_-1.5kb | 20 | 0.7685 ± 0.03 |  |  |  |  |
| ch4_-4.5kb | 9 | 0.6671 ± 0.02 |  | ch8_-0.132kb | 5 | 0.4636 ± 0.02 |  | ch3_335kb | 31 | 0.9413 ± 0.00 |
| ch4_-4.4kb | 15 | 0.8108 ± 0.01 |  | ch8_0.034kb | 23 | 0.8273 ± 0.02 |  | ch3_363kb | 28 | 0.9231 ± 0.01 |
| ch4_-3.8kb | 9 | 0.7230 ± 0.02 |  | ch8_0.5kb | 7 | 0.5919 ± 0.03 |  | ch3_383kb | 27 | 0.8588 ± 0.02 |
| ch4_-1.2kb | 13 | 0.7997 ± 0.01 |  | ch8_1.4kb | 24 | 0.6871 ± 0.04 |  | ch3_429kb | 24 | 0.8778 ± 0.01 |
| ch4_-0.30kb | 8 | 0.4409 ± 0.04 |  | ch8_6.4kb | 21 | 0.8173 ± 0.02 |  | mean | 27.5 | 0.9003 |
| ch4_0.20kb | 9 | 0.5113 ± 0.03 |  | ch8_9kb | 17 | 0.8187 ± 0.02 |  |  |  |  |
| ch4_0.52kb | 15 | 0.5782 ± 0.03 |  | ch8_16.3kb | 40 | 0.9350 ± 0.01 |  | mean (ch 2 & 3) | 26.45 | 0.9071 |
| ch4_1.48kb | 2 | 0.2864 ± 0.03 |  | ch8_22.8kb | 16 | 0.8120 ± 0.02 |  |  |  |  |
| ch4_4.05kb | 17 | 0.8167 ± 0.02 |  | ch8_36kb | 18 | 0.8734 ± 0.02 |  |  |  |  |
| ch4_5.87kb | 11 | 0.8048 ± 0.01 |  | ch8_49.5kb | 24 | 0.8734 ± 0.01 |  |  |  |  |
| ch4_30.31kb | 26 | 0.9299 ± 0.01 |  | ch8_66.1kb | 22 | 0.9324 ± 0.00 |  |  |  |  |
| ch4_49.16kb | 31 | 0.9532 ± 0.01 |  |  |  |  |  |  |  |  |
|  |  |  |  |  |  |  |  |  |  |  |
