# Supplementary material for: Differences in selective pressure on dhps and dhfr drug resistant mutations in western Kenya
Source: Malar J. 2012 Mar 22;11:77. doi: 10.1186/1475-2875-11-77 (PMC3338400; doi:10.1186/1475-2875-11-77)
Supplement: Additional file 8 — Figure S6. Observed and predicted He at ms loci around the (A) dhfr allele 51I/59R/108N and (B) dhps allele 437G/540E. For the dhfr prediction (A) we used He among all samples that did not include the 51I/59R/108N triple mutant (i.e. infections with the mixed codons N51I/S108N and C59R/S108N were included). For the dhps prediction (B) we used He among wildtype alleles as an estimate for the initial heterozygosity. Loci are labelled according to their positions relative to dhfr or dhps (kb from the gene). Sampling variance is indicated by error bars. [file 1475-2875-11-77-S8.DOC]

**A**

**B**

Figure 6S Observed and predicted *H_e_* at ms loci around the (A) *dhfr* allele 51I/59R/108N and (B) *dhps* allele 437G/540E. For the *dhfr* prediction (A) we used *H_e_* among all samples that did not include the 51I/59R/108N triple mutant (i.e. infections with the mixed codons N51I/S108N and C59R/S108N were included). For the *dhps* prediction (B) we used *H_e_* among wildtype alleles as an estimate for the initial heterozygosity. Loci are labelled according to their positions relative to *dhfr* or *dhps* (kb from the gene). Sampling variance is indicated by error bars.
