# Supplementary material for: Differences in selective pressure on dhps and dhfr drug resistant mutations in western Kenya
Source: Malar J. 2012 Mar 22;11:77. doi: 10.1186/1475-2875-11-77 (PMC3338400; doi:10.1186/1475-2875-11-77)
Supplement: Additional file 9 — Figure S7. Pairwise LD between microsatellite loci on different chromosomes (A) and between sites in dhfr and dhps (B). Each cell represents one comparison between polymorphic pairs of loci. Gray cells represent significance at p value < 0.01. (A) The position of dhfr and dhps along the chromosome is denoted by the gray bar. The location of each microsatellite locus is at the top of the matrix (loci are named according to their positions relative to dhfr or dhps or along chromosome 2 or 3 according to the 3D7 genome sequence available from NCBI). (B) Pairwise LD between sites in dhfr (51, 59, 108) and dhps (436, 437, 540). [file 1475-2875-11-77-S9.DOC]

A


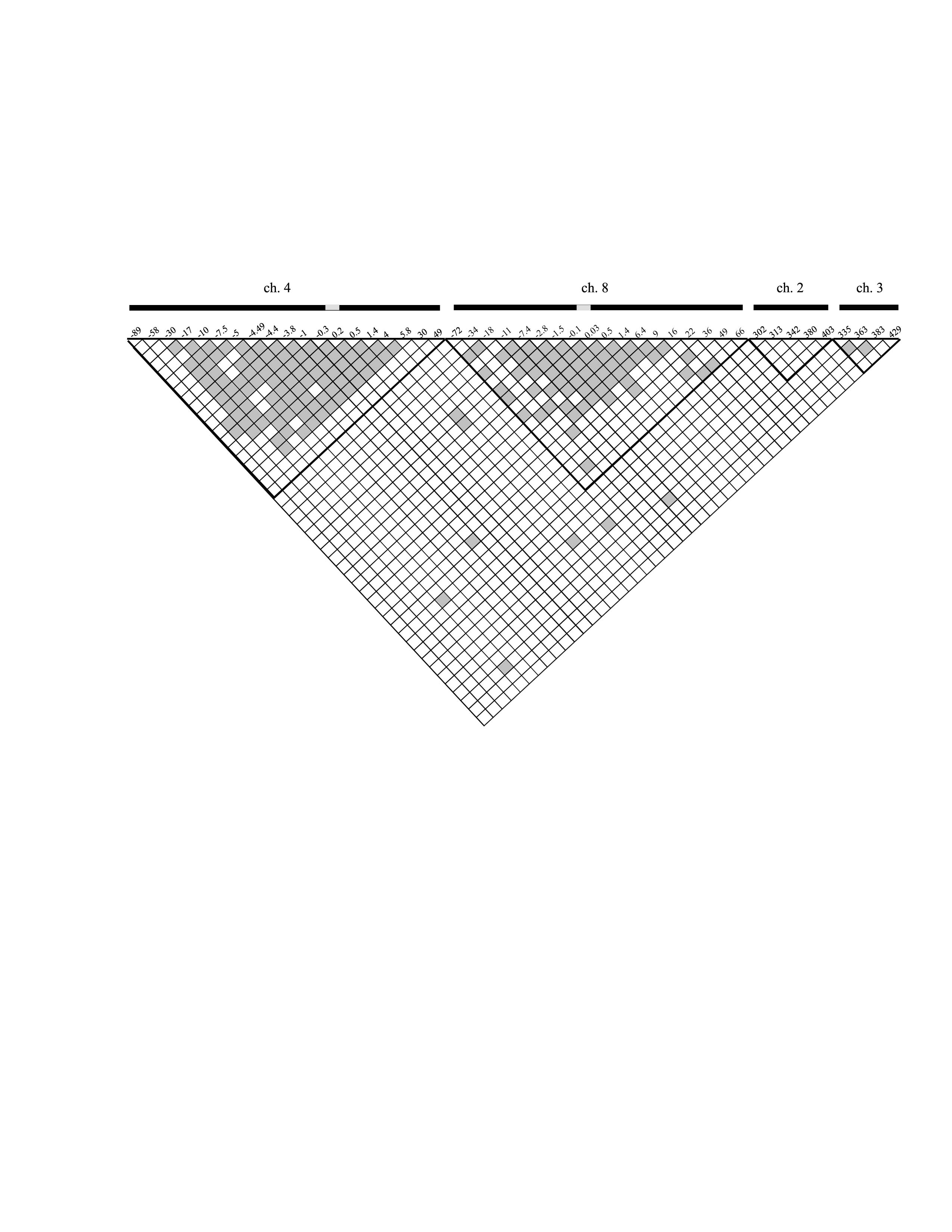


B

|  | 51 | 59 | 108 | 436 | 437 | 540 |
| --- | --- | --- | --- | --- | --- | --- |
| 51 | - |  |  |  |  |  |
| 59 |  | - |  |  |  |  |
| 108 |  |  | - |  |  |  |
| 436 |  |  |  | - |  |  |
| 437 |  |  |  |  | - |  |
| 540 |  |  |  |  |  | - |

Figure 7S. Pairwise LD between microsatellite loci on different chromosomes (A) and between sites in *dhfr* and *dhps* (B). Each cell represents one comparison between polymorphic pairs of loci. Gray cells represent significance at p value<0.01. (A) The position of *dhfr* and *dhps* along the chromosome is denoted by the gray bar. The location of each microsatellite locus is at the top of the matrix (loci are named according to their positions relative to *dhfr* or *dhps* or along chromosome 2 or 3 according to the 3D7 genome sequence available from NCBI). (B) Pairwise LD between sites in *dhfr* (51, 59, 108) and *dhps* (436, 437, 540).
